# A 26-year time series of mortality and growth of the Pacific oyster *C. gigas* recorded along French coasts

**DOI:** 10.1038/s41597-022-01511-2

**Published:** 2022-07-09

**Authors:** Anna Mazaleyrat, Julien Normand, Laurent Dubroca, Elodie Fleury

**Affiliations:** 1 Laboratoire de Biologie des Organismes et Ecosystèmes Aquatiques (BOREA) Université de Caen-Normandie, MNHN, SU, UA, CNRS, IRD, Esplanade de la Paix – CS, 14032 CAEN Cedex 5, France; 2 Ifremer, LERN, F-14520 Port-en-Bessin, France; 3 Ifremer, LRHPB, F-14520 Port-en-Bessin, France; 4 Univ Brest, Ifremer, CNRS, IRD, LEMAR, F-29280 Plouzané, France

**Keywords:** Ecological modelling, Time series, Standardization, Marine biology, Biooceanography

## Abstract

We used a compiled data set from a monitoring network of oyster production coordinated by IFREMER (the French Research Institute for the Exploitation of the Sea). This network monitors the growth and mortality of the Pacific oyster *Crassostrea gigas* along French coasts since 1993. The archive, although publicly available, has been challenging to use due to changes in protocols and little information on metadata. Here, we describe data collection for almost 30 years, cleaning and processing. For 13 locations, we modeled growth and mortality of spat (less than one-year-old individuals) and half-grown oysters (between one and two-year-old individuals) as a function of time to cope with changes in data acquisition frequency, and produced standardized annual growth and cumulative mortality indicators to improve data usability. This improved database is expected to be used by ecologists interested in the evolution of life-cycle indicators of a marine species under the influence of climate change. It can also be valuable for epidemiologists because mortality data traces the emergence and spread of a massive epizootic.

## Abstract (summary)

**Table:** 

Measurement(s)	body mass • Cumulative mortality
Technology Type(s)	balance • Count
Factor Type(s)	Location • Sampling year
Sample Characteristic - Organism	Crassostrea gigas
Sample Characteristic - Environment	sea coast
Sample Characteristic - Location	Metropolitan France

## Background & Summary

In the last two decades, a consensus emerged that human activities had become the main drivers of ecosystems functioning^1^. At the land-sea interface, estuarine and coastal ecosystems are particularly affected by climate change and more direct anthropogenic pressures such as coastal engineering (habitat alteration) and pollution^2^. In their recent review, Cloern *et al*.^3^ reported rapid changes in species communities and abrupt fluctuations of productivity of estuarine–coastal ecosystems in different contexts. However, some authors also pointed out that these variations are difficult to interpret, since the fluctuations of abiotic factors such as salinity, temperature, nutrients and oxygen concentrations vary simultaneously, often in an erratic way^4^. It, therefore, seems crucial to disentangle short-term variations from long-term trends and human-induced alterations from “natural” evolution to determine the processes that drive such fluctuations of ecosystems functioning. Monitoring programs conducted over decades and across a large spatial scale provide valuable data for assessing the state and the pressures affecting the ecosystems^5,6^. Unfortunately, very few long-term datasets on marine ecosystems have been released (but see: Ocean Biodiversity Information System [OBIS: https://obis.org] and European Marine Observation and Data Network [EMODnet: https://www.emodnet-biology.eu/]).

Filter feeders play a crucial role in building reef habitats and trophic resources and are thus considered ecosystem engineers. Among them, the Pacific oyster, *Crassostrea gigas*, also represents economic value through aquaculture. In the late sixties, this species, native to the NW Pacific and the Sea of Japan, has been massively introduced worldwide. In France, this species was brought by local oyster farmers to replace the Portuguese oyster (*C. angulata*) affected by a viral disease^7,8^. In the late eighties, significant mortality events of *C. gigas* occurred in France^9^, one of the main producers of oysters in Europe^10^, and particular interests in biological monitoring of *C. gigas* emerged. The causes of mass mortality events seem to differ between spat and half-grown oysters^11^. Indeed, mortality events of spat are mainly caused by Ostreid herpesvirus type 1 (OsHV-1)^12^, whereas those of half-grown oysters are caused by strains of the *Vibrio aestuarianus* bacteria. In the two diseases, pathogens interact with the host and environmental risk factors to determine the dynamic and the severity of the outbreak^11,13–17^. Considering the complexity of the different processes that influence mortality proportions, monitoring made it possible to disentangle the drivers of oyster outbreaks in the field^11,15,18^.

Here, we took advantage of a monitoring network of Pacific oyster production implemented along French coasts since 1993 and coordinated by IFREMER to build a consolidated open-source database of oyster growth (*i.e*. the changes in mass over time) and mortality. This network has evolved over the years and encompasses three monitoring programs: REMORA (monitoring of mollusks production yields between 1993 and 2009), RESCO (observation network of bivalve mollusks from 2009 to 2014), and finally ECOSCOPA (national French observatory of the life-cycle of the Pacific oyster, since 2015). Although these programs had different objectives, REMORA focused on the monitoring of rearing performances across sites and years, RESCO aimed at monitoring oyster health with high frequency in an epizootic context, while ECOSCOPA used sentinel oysters to assess environmental variations, they all collected observations relative to oyster growth and mortality. They also operated on common sites, located in a wide range of environments from the Mediterranean Thau lagoon to the estuarine Baie des Veys in the English Channel. Raw data from these three programs are already made available in a SEANOE database^19^. However, because the objectives differed among these programs, data frequency acquisition and protocols varied through time, and the metainformation relative to these changes has not been fully consolidated yet. The result is that the use of raw data by an external user appears very tricky. Although an exhaustive list of all studies using these data seems unrealistic, a recent bibliographical review revealed that 82 articles published between 2008 and 2021 cited the French oyster larvae monitoring network (VELYGER: https://www.seanoe.org/data/00308/41888/) or/and the monitoring network of Pacific oyster production^20^. To our knowledge, the data from the monitoring network of Pacific oyster production have mainly been used in a research context to investigate the risk factors associated with mortality events^15,21–23^, the variability of oyster growth^24^, and to model the evolution of the invasive potential under contrasted climate scenarios^21,23^.

To extend the reuse potential of these data, we extracted and aggregated from this database the information relevant for quantifying the variations in growth and mortality of oysters across sites and years. We then modeled the evolution of growth and mortality as a function of time to cope with changes in data frequency acquisition and computed standardized indicators. This database may help to quantify the effect of environmental variations on life-history traits of a marine species and forecast simulations of *C. gigas* traits under climate change scenarios (*e.g*. for reproductive traits^21,25^ and for mortality occurence^23^). It may also be of interest for epidemiologists because mortality data traces the emergence and spread of a massive epizootic in *C. gigas* spat.

## Methods

### Experimental design

**Fig. 1:**
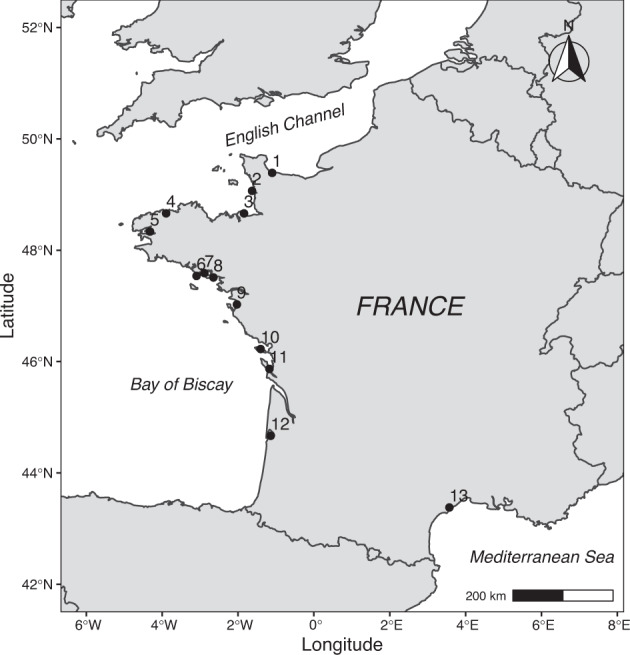
Site locations (coordinates in WGS84) along the French coastline. The site numbers refer to Table 1.

Data collection took place in different sites disseminated along the mainland French coastline in sectors dedicated to Pacific oyster farming. Over the years, the number of sites monitored varied from 43 sites until 2009, to 13 between 2009 and 2013, and finally to 8 sites since 2015. Here, we focus on 13 sites (Fig. 1 & Table 1) that were almost continuously monitored since 1993. All these sites stand in tidal areas except Marseillan, located in the Mediterranean Thau lagoon, for which tidal variations are only tenuous and Men-er-Roué which is in subtidal deep-water oyster culture area in the Bay of Quiberon. Sentinel oysters were reared in plastic meshed bags fixed on iron tables, mimicking the oyster farmers practices. In Marseillan, half-grown oysters were cemented onto vertical ropes (from 1993 to 2007 and from 2015 to 2018), reared in Australian baskets (from 2008 to 2011), or put in bags fixed on iron tables (2012, 2013, 2014). As for spat oysters, they were reared in pearl-nets between 2008 and 2011 or put in bags since 2012.

**Table 1:** Site identification and coordinates in WGS84.

Site number	Name	Zone	Latitude	Longitude
1	Géfosse	Bay of Seine	49.389150	−1.099767
2	Blainville-sur-mer	West Cotentin Peninsula coast	49.065780	−1.629950
3	Cancale	Bay of Mont Saint-Michel	48.660980	−1.841353
4	Morlaix	Bay of Morlaix	48.662340	−3.895002
5	Pointe du Château	Bay of Brest	48.335000	−4.319390
6	Men-er-Roué	Bay of Quiberon	47.538160	−3.093013
7	Larmor-Baden	Morbihan Gulf	47.588460	−2.885802
8	Pénerf	Pénerf River	47.510110	−2.648004
9	Coupelasse	Bay of Bourgneuf	47.026020	−2.030078
10	Loix-en-Ré	Pertuis Breton/Ré Island	46.225070	−1.404059
11	Banc d’Agnas	Pertuis d’Antioche/Bay of Marennes-Oléron	45.868540	−1.172305
12	Le Tès	Bay of Arcachon	44.665950	−1.138744
13	Marseillan	Thau Lagoon	43.379130	3.571080

During the 1993–2013 period, at the beginning of each annual campaign, one batch of diploid spat (three in 2012 and 2013) and one batch of diploid half-grown oysters were bought from an oyster farmer (*i.e*., wild-caught individuals) and then deployed simultaneously on all sites of the monitoring network. Here, the term “batch” designates a group of oysters born from the same reproductive event (spatfall or hatchery cohort), having experienced strictly the same zootechnical route. One batch could eventually be reared in several different bags (up to 3) deployed in the same site. Different batches were never mixed in the same bag.

During the 2009–2013 period, up to three additional batches of triploid spat were bought in commercial hatcheries and included in the survey strategy (for a maximum of 6 batches of spat per site in 2012 and 2013). In 2009, the batches that were bought had already been exposed to a first wave of mortality before being followed by the network. Thus, the data collected this year should be interpreted with caution. Since 2014, the origin of spat and half-grown oysters has changed notably to better control the initial health status of oysters (no contact with the natural environment before deployment in all sites). The hatchery facility of Ifremer-Argenton now produces the sentinel diploid spat used in the monitoring network (one batch for all sites per campaign), whereas, the half-grown oysters was composed of spat reared on the same location the previous year but not monitored.

### Data collection

After the deployment of the different batches at the beginning of the campaign (seeding dates from February to April depending on the year), growth and mortality were longitudinally monitored yearly. Until 1999, annual campaigns usually ended in the winter of the year the monitoring began (*i.e*. in December), whereas, during the period 2000–2018, all sites frequently extended the campaign to end in the winter (February to March) of the following year.

Observations were collected on each site quarterly until 2008 but then monthly to bimonthly depending on the season. At each sampling date, local operators carefully emptied each bag in separate baskets, counted the dead individuals (those with open or empty shells) and alive ones, and removed the dead individuals. Then local operators weighed all alive individuals in each basket (mass taken at the bag level, protocol mainly used between 1993 and 1998 and since 2004) and/or collected 30 individuals to individually weigh them in the laboratory (mass taken at the individual level, protocol used between 1995 and 2010 for spat and since 1996 for half-grown oysters).

### Data cleaning

During the 2009–2013 period, several batches of spat were monitored per site and campaign. Some had a similar background to the batches monitored before 2009 (*i.e*., wild-caught spat from natural spatfall collected in the bay of Arcachon). To ensure the continuity of the time-series, we thus decided to remove all mass and mortality data of spat that did not originate from natural spatfall in the Bay of Arcachon, as well as triploid spat bought in hatcheries (see Table 2 for the origin and number of batches kept per site and campaign). To ensure that the life-cycle indicators are as comparable as possible between campaign and site (*i.e*. estimated in a common restricted time window), we removed data collected after December 31 of the year the monitoring began, as well as the site × campaign combinations when monitoring ended before October because the growth or mortality could still be in the exponential phase during this end-of-follow-up periods^26^. As the protocol of mass data collection changed over the years, we could not only use the mass data taken at the bag level or that at the individual level without greatly breaking the continuity of the time-series. We thus kept data taken at the individual level until 2008 and those taken at the bag level since 2009. We then checked for nonsense or missing data (*e.g*., the mass of a bag was equal to 0 or missing although they were still alive oysters in the bag), duplicated values and removed data for bags not part of the protocols or incorrectly identified. Finally, we removed site × campaign combinations for which we had fewer than four mass or mortality data because more data is necessary to study the temporal pattern of growth and mortality.

**Table 2:** Origins of the different oyster batches retained after data cleaning.

Different colors indicate different strategies concerning the distribution of oyster batches and the origin of spat.

### Data processing and analysis

At this point, the available data were, therefore, the number of living individuals per bag, the number of dead individuals since the last visit, the individual mass (g) of oysters (until 2008) and the total mass (g) of the living individuals per bag (since 2009).

For mass data collected until 2008, we calculated the mean of the individual mass per date × site × age class combination by averaging the mass of the individuals. In other cases (mass data collected since 2009), we calculated the mean mass of individuals for each bag × date × site × age class combination by dividing the total mass of living oysters by the number of living individuals and then averaged data by date × site × age class combination. Our mass data, hereafter called mean mass data, is thus composed of the mean of the individual mass until 2008 and the mean mass of individuals since 2009.

For mortality data, we could not calculate a cumulative mortality per bag × date × site × age class combination as $1-\frac{number\;of\;alive\;oysters\;at\;sampling\;date}{number\;of\;oysters\;at\;previous\;sampling\;date}$ because the total number of oysters (dead and alive) on a specific date often differed from the number of alive oysters at the previous date (*e.g*., because oysters were lost from the bags, or were sampled for complementary analyses such as pathogen detection). We thus took into account changes in oyster numbers between visits and calculated cumulative mortality using the following formula: *CM*_*t*_ = 1 − ((1 − *CM*_*t-1*_) × (1 − *IM*_*t*_)). *CM*_*t*_ = Cumulative mortality at time *t*; *CM*_*t-1*_ = Cumulative mortality at time *t*-1; *IM*_*t*_ = Mortality rate at time *t*. *IM*_*t*_ was obtained by dividing the number of dead oysters by the sum of alive and dead oysters at time *t*. When several bags were followed, we then averaged the cumulative mortality per date × site × age class combinations.

We modeled the evolution of the mean mass and cumulative mortality data as a function of time to cope with changes in data frequency acquisition during annual monitoring campaigns. According to previous studies, annual mortality and growth curves in *C. gigas* follow a sigmoid curve^11,26^. Therefore, we fitted a logistic model, Eq. (1), and a Gompertz model, Eq. (2), which correspond to the most commonly used sigmoid models for growth and other data^27^, to describe *Y*_*t*_ = mean mass (in grams) and cumulative mortality at time *t*.

1 $${Y}_{t}=\frac{a}{\left(1+{e}^{\left(-b\times \left(t-c\right)\right)}\right)}$$

2 $${Y}_{t}=a\times {e}^{\left(-e\left(-b\times \left(t-c\right)\right)\right)}$$

These equations estimate three parameters: the upper asymptote (a), the slope at inflection (b), and the time of inflection (c).

As the mean mass of half-grown individuals at the beginning of the campaign was higher than 0, we also fitted a four-parameter version of the logistic model, Eq. (3), and Gompertz model, Eq. (4), which is commonplace in the growth-curve analysis of bacterial counts^27^, and estimated (d) which represents the lowest asymptote of the curve. This parameter also moves the model curve vertically without changing its shape. The upper asymptote thus becomes equal to d + a.

3 $${Y}_{t}=d+\frac{a}{\left(1+{e}^{\left(-b\times \left(t-c\right)\right)}\right)}$$

4 $${Y}_{t}=d+a\times {e}^{\left(-e\left(-b\times \left(t-c\right)\right)\right)}$$

Model fitting was carried out using non-linear least squares regressions (R package *nls.multstrat*^28^). This method allows running 5000 iterations of the fitting process with start parameters drawn from a uniform distribution and retaining the fit with the lowest score of Akaike Information Criterion (AIC). The sigmoid curve (*i.e*. logistic or Gompertz) with the lowest mean AIC of all models was selected as the best curve describing the data (see *technical validation* section).

## Data Records

We provide four data sets. The first data set contains the raw observations of oyster mortality and growth recorded within the REMORA, RESCO and ECOSCOPA programs. The second data set is the clean data set of oyster growth and mortality. It contains the calculated cumulative mortality and mass (g) of spat and half-grown oysters in 13 sites across the French coastline for 26 years. One row corresponds to the mean cumulative mortality and the mean mass of oysters for a specific date × site × age class combination. The third data set contains the mass (g) and cumulative mortality predicted by the best sigmoid model for each day × campaign × site × age class combination. The predictions range from the Day Of the Year (DOY) 65 (median day of seeding date) to DOY 337 (median day of the end of monitoring). The last data set contains information about the sites (e.g., coordinates). All these datasets are publicly available on the data depository Zenodo^29^ where a full description of each column and its units is provided to extend their reuse potential. It is noteworthy to mention that the first data set already has a digital object identifier associated with the SEANOE database^19^, which is regularly updated with new data from the monitoring network. It is therefore very likely that other repositories will be made in the coming years, and that these new repositories will be linked to the same DOI. The architecture of this database is also likely to evolve (e.g., change in name or column order) from one repository to the next. These changes are the result of several adjustments to the database architecture made over the years, but also of the formulation of the query sent to the original database hosted in the Quadrige^2^ information system developed by IFREMER. These changes in the database architecture and content, combined with the fact that only the latest repository is fully accessible, imply that we cannot guarantee the reproducibility of this work by providing only the dataset DOI. We, therefore, hosted the raw database extraction on Zenodo^29^ to comply with the FAIR guidelines^30^.

**Fig. 2:**
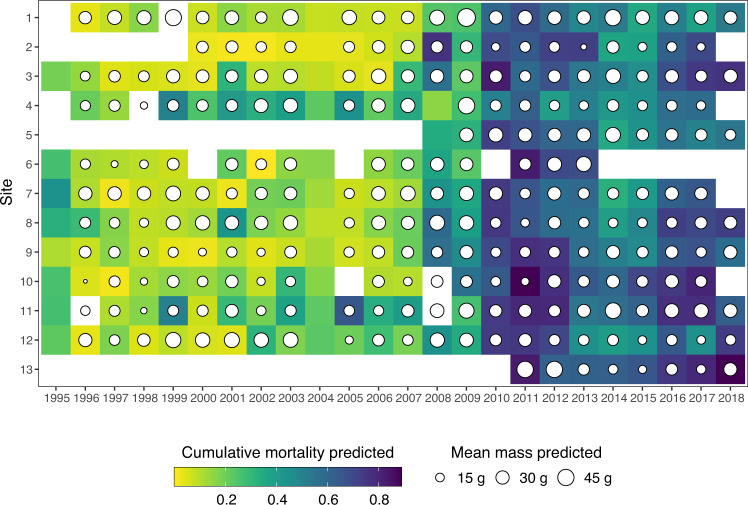
Cumulative mortality and mean mass predicted on DOY 337 (median day of the end of the monitoring) for spat across 24 campaigns and 13 sites. Empty cells symbolize data not available or removed from the analysis. The site numbers refer to Table 1.

**Fig. 3:**
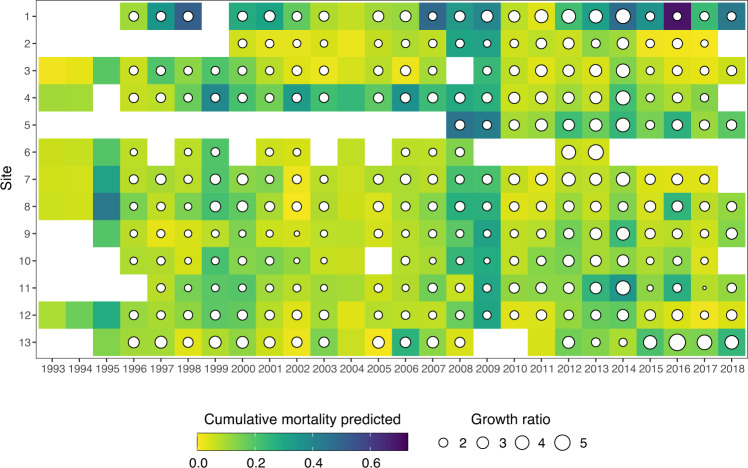
Cumulative mortality predicted on DOY 337 (median day of the end of the monitoring) and growth ratio predicted expressed as the mean mass on DOY 337 divided by the mean mass on DOY 65 (median day of seeding date) for half-grown oysters across 26 campaigns and 13 sites. Empty cells symbolize data not available or removed from the analysis. The site numbers refer to Table 1.

**Fig. 4:**
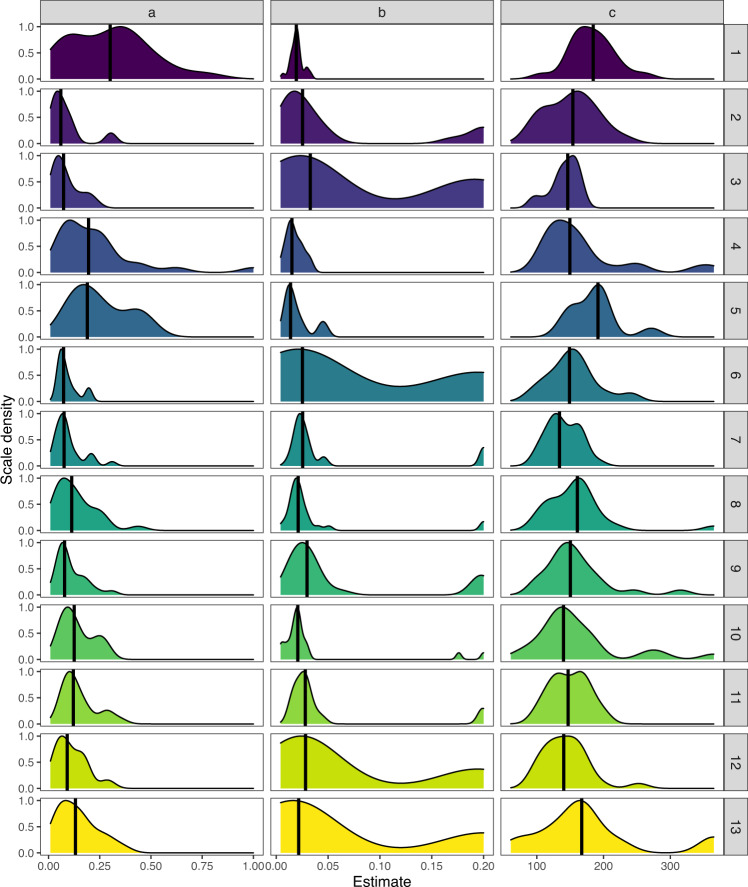
Distribution of the estimates (**a**,**b**,**c**) extracted from the Gompertz models predicting the cumulative mortality of half-grown oysters. The site numbers refer to Table 1. The vertical line represents the median.

Changes in predicted mean mass and cumulative mortality for spat and half-grown oysters across the campaign and site are shown in Figs. 2 and 3. Predicted cumulative mortality for spat on DOY 337 (median day of the end of the monitoring) ranges from 0 to 0.9 and has strongly increased since 2008 (Fig. 2), whereas cumulative mortality for half-grown oysters varies between 0 and 0.7 (Fig. 3). As for the predicted mean mass, it ranges from 9 g to 54 g for spat and was multiplied up to almost 6 times for half-grown oysters between DOY 65 (median day of seeding date) and DOY 337. These values are consistent with previous literature^31,32^. The parameters estimated in the Logistic and Gompertz models (Fig. 4) can also be valuable for ecologists as they may be compared between species or within species in different ecosystems.

## Technical Validation

To maintain data quality and ensure that the protocol changes do not prevent the comparisons of mass or mortality from one year to another, several elements have been verified.

We first determined whether changes in the origin of spat (*i.e*. from natural spatfall or produced in the hatchery) could affect the mass and mortality indicators. To do so, a batch of wild-caught spat collected in the bay of Arcachon (*i.e*. a batch with a similar background to that of the one deployed until 2014) and a batch of spat produced in the IFREMER hatchery were deployed in 2014 in all sites. Comparisons between these batches show that mortality peaks simultaneously occurred in the two batches and that mortality rates at specific dates were comparable^33^. Monitoring of batches of wild-caught spat from natural spatfall was thus abandoned in 2014 and spat batches are now produced in the IFREMER hatchery through a standardized protocol. This protocol minimizes the risk that the animals used carry pathogens before monitoring by performing a heat test and analysis to detect OsHV-1 DNA^34^ (a virus often involved in mortality outbreaks of spat). This standardized spat (free from OsHV-1, any pathogens notifiable, and any abnormal mortality during the breeding cycle) makes it possible to study more precisely the annual variation of oyster’s growth and cumulative mortality and the effects of changes in environmental conditions on oysters by controlling their initial health status and minimizing the variability between batches.

Second, we verified whether using two methods to calculate the mean mass of oysters could bias our indicators. To do so, a comparison of the mean of individual mass (mass taken at the individual level) collected in all sites in 2014 to the mean mass of individuals (mass taken at the bag level divided by the number of alive individuals) was realized. Results revealed no significant difference in the calculated mean whether it was obtained via mass level data or individual level data (Pepin and Durand, *comm pers*). Using the mean of individual mass until 2008 and the mean mass of individuals since 2009 is thus suitable.

**Fig. 5:**
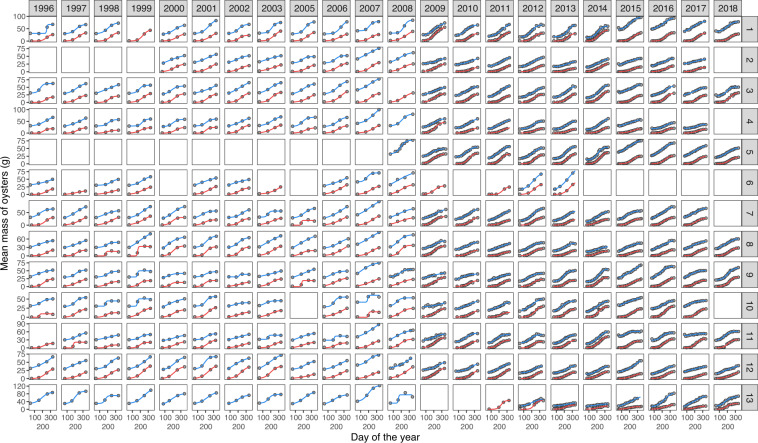
Mean mass of oysters calculated () and predicted for spat () and half-grown oysters () as a function of time for 22 campaigns and 13 sites. The lines represent the best fitting model (*i.e*. a logistic one for spat and half-grown oysters). The empty squares symbolize the absence of data. The site numbers refer to Table 1.

**Fig. 6:**
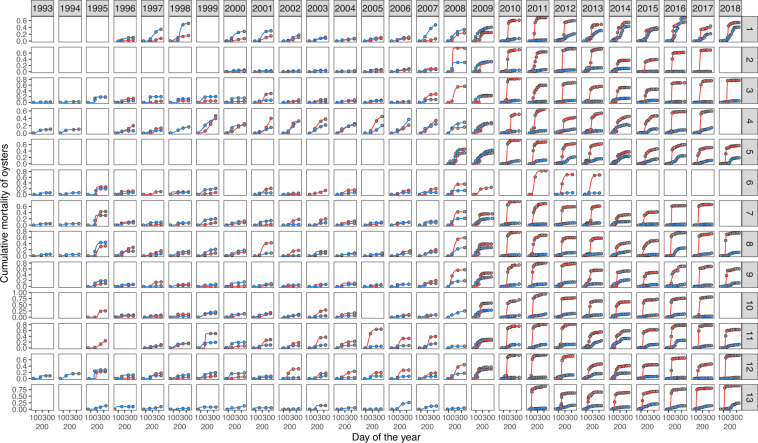
Cumulative mortality of oysters calculated () and predicted for spat () and half-grown oysters () across 26 campaigns and 13 sites. The lines represent the best fitting model (*i.e*. a Gompertz model for both spat and half-grown oysters). The empty squares symbolize the absence of data. The site numbers refer to Table 1.

**Fig. 7:**
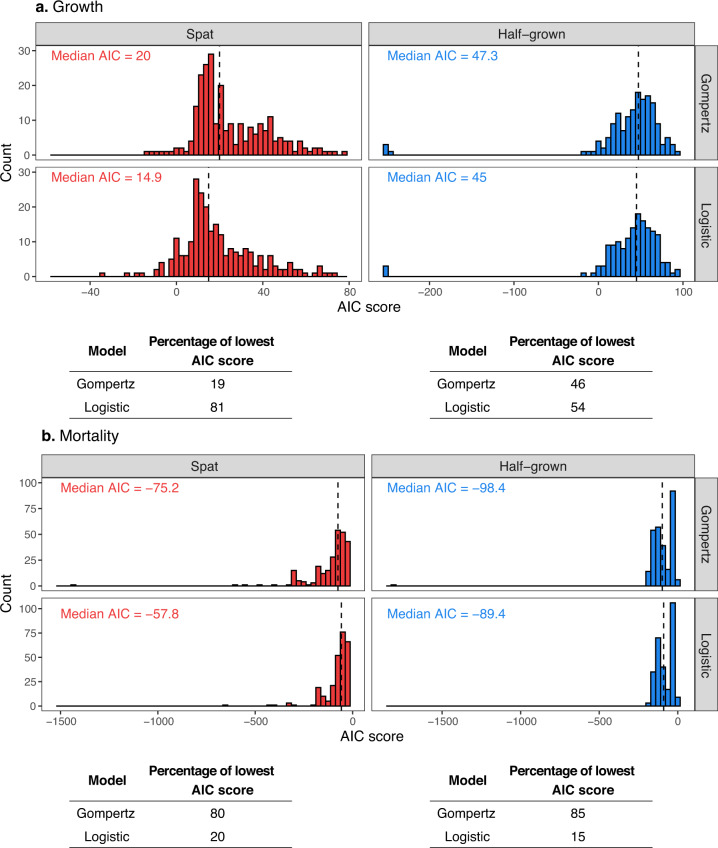
Distribution of AIC scores for the Gompertz and logistic models fitted to spat () and half-grown () growth data (**a**) and cumulative mortality data (**b**). We fitted a logistic and a Gompertz model to growth and cumulative mortality curves for each campaign × site × age class combination. The Akaike’s Information Criterion scores (AIC) for each model was calculated and compared between models (logistic and Gompertz) for each age class to select the best model. The tables indicate which of the model had the lowest AIC.

Finally, good fit of growth (Fig. 5) and mortality curves (Fig. 6) were visually inspected and compared with AIC. The logistic model yielded the lowest AIC for the mass data of spat and half-grown oysters (Fig. 7a), whereas the Gompertz model best fitted the cumulative mortality data (Fig. 7b).

## Data Availability

All figures have been produced using R (version 4.1.2) and RStudio (version 2021.09.1 + 372). The scripts used are available in a GitHub repository^35^ and are archived on Zenodo^29^.
